# The Demon of Chloral

**Published:** 1882-03

**Authors:** 


### THE DEMON OF CHLORAL.

A woman who would have shuddered at the idea of drinking too much wine, thinks little of taking repeated doses of chloral—the small, innocent-looking vial so constantly in demand that it is thought nothing of. And yet the brandy bottle may be the safer of the two. We see a woman lolling languidly in her carriage, in her box at the opera, or even at her own table, with a filmy, dazed look in her eyes, an absent manner, and a half unconscious way of listening and replying to those about her. We at once account for it: "Chloral."

We see a man who was once the incarnation of life and energy, brisk in business, intellectual, cultivated. We see him dulled, nerveless, his brighter self effaced, his intellect flickering like a dying light, his business faculties dwindling, slovenly in mind as well as person, inaccurate in statements and careless as to linen, and we say, "Chloral."

There are, in our merry England, beings who are as wholly under the dominations of morphia as ever was Chinese under that of opium. Women have yielded by degrees to its fatal fascination until at last they prick the skin a dozen times a day with the tiny syringe that has such terrible results.

The operation is almost painless; the immediate effects pleasant. A delicious languor supervenes. Happy thoughts, bright imaginings fill the mind. Some see beautiful visions; others feel only a pervading comfort and well-being. But at the very height of the enchantment, the influence of the morphia begins to subside. The sensation of comfort gives place to one of discomfort, irritation, even pain. And the end? The punishment is terrible indeed. By degrees the mind becomes darkened. Hideous hallucinations seize upon it. Self-control is lost. Imbecility overtakes the weak. Madness threatens the strong. These are the personal consequences.

There are others to be bequeathed to sons and daughters and to later generations. These can be guessed at. The new vice has not reigned sufficiently long for the world to have seen them exemplified; but a dark array of possibilities suggests itself but too readily. The heritage of insanity, of inebriety, of imbecility, will in future days be traced back to those tiny tubes which hold but a drop or two, and to which men once looked as to a blessed means of relieving pain, forgetting that blessings and cures go hand in hand in a crooked world. Dipsomania has now a powerful rival, speedier in its results than its own revolting process, and eventually as degrading. The name of the later-born sister-fiend is Morphiomania.—*London Truth.*

